# Circulating Insulin and IGF-1 and Frequency of Food Consumption during Pregnancy as Predictors of Birth Weight and Length

**DOI:** 10.3390/nu13072344

**Published:** 2021-07-09

**Authors:** Dominika Mazurkiewicz, Monika Bronkowska

**Affiliations:** Department of Human Nutrition, Faculty of Biotechnology and Food Sciences, Wrocław University of Environmental and Life Sciences, ul. Chełmońskiego 37, 51-630 Wrocław, Poland; monika.bronkowska@upwr.edu.pl

**Keywords:** insulin, IGF-1, Food Frequency Questionnaire (FFQ), weight gain, birth weight, birth length, Ponderal index

## Abstract

The aim of the study was to assess the relationships between maternal insulin and insulin-like growth factor-1 (IGF-1) concentration and food consumption frequency and the birth parameters of the newborn. A total of 157 mother-newborn pairs participated in the study. The study showed that more frequent consumption of sweet and salty snacks as well as fruit and fruit or vegetable juices may promote greater weight gain in pregnancy and higher newborn birth weight. A significantly higher insulin concentration was found among overweight women according to body mass index (BMI), and a significantly lower concentration of IGF-1 was demonstrated among women ≥35 years of age. There was no significant correlation between the concentration of insulin and IGF-1 in the mother’s blood plasma and the birth weight and length of the newborn. A significant relationship was only found between the concentration of IGF-1 in the mother’s blood and the Ponderal index of the newborn. A woman’s eating habits during pregnancy have a significant impact on the mother’s health and on the proper growth and development of the foetus.

## 1. Introduction

Maternal nutritional status and metabolic factors have a significant effect on foetal growth and development and thus determine the health of the newborn. Maternal malnutrition during pregnancy or a body mass index (BMI) below normal before pregnancy may cause abnormal foetal development or low neonatal birth weight (<2500 g). A woman’s obesity before pregnancy, excessive weight gain during pregnancy, or a suboptimal diet composition (high intake of simple sugars or saturated fatty acids) may contribute to high birth weight (>4000 g), the occurrence of metabolic disorders, and higher childhood body fat in the children of such women compared with those of non-obese women [1,2,3].

Pregnancy is a period characterized by numerous metabolic adaptation processes, including an increase in insulin and insulin-like growth factor (IGF) concentrations and an increased pancreatic beta-cell response. Insulin and IGF are involved in the regulation of placental and foetal growth and development. The main function of insulin is the regulation and modulation of metabolic processes and transport of nutrients across the placenta, e.g., glucose and fatty acids. Various IGFs (IGF-1 and IGF-2) are involved in increased glucose uptake, amino acid transport across the placenta, and foetal or placental cell proliferation. The metabolic changes occurring in the woman’s body are to maintain the proper nutritional status of both the mother and the foetus and to prepare the mother’s body for childbirth and breastfeeding [4,5]. During pregnancy, insulin resistance in the cells of the woman’s body develops mainly in the second and third trimesters. The increase in insulin concentration and insulin resistance in pregnancy is a biological mechanism designed to provide transport of glucose to the foetus [4,5,6]. The occurrence and level of insulin resistance is modified by many factors, e.g., hormones, cytokines, obesity, and diet. Obesity before pregnancy or excessive weight gain in pregnancy may contribute to increased insulin resistance and gestational diabetes, which may lead to complications in foetal development and perinatal conditions. Maternal chronic hyperglycaemia (caused by incorrect eating habits) could leads to gestational diabetes during pregnancy and could cause foetal hyperglycaemia, hyperinsulinemia, or macrosomia [4,7]. The growth of adipose tissue causes increased synthesis of adipokines and cytokines, such as leptin, adiponectin, tumour necrosis factor alpha (TNF-alpha), or interleukin-6 (IL-6). Additionally, the placenta synthesizes factors that may increase insulin and insulin resistance in the mother’s body. Insulin sensitivity is reduced by the increased concentration of oestrogens, cortisol, leptin, glucagon, TNF-alpha, and placental hormones, including human placental lactogen (hPL). The increased insulin secretion is caused by oestrogens, TNF-alfa, and hPL. Additionally, the hPL is involved in beta-cell proliferation during pregnancy and stimulates maternal production of IGF-1 [4,7,8].

The aim of the study was to assess the relationship between maternal insulin and IGF-1 concentration as well as food consumption frequency and birth parameters of the newborn. The novel approach is that the study presented in the manuscript concerns women with a physiological pregnancy without disturbances in carbohydrate metabolism during pregnancy; it is also a multifactorial approach because it links the concentration of selected hormones with the nutritional status of the mother, her diet, and the birth parameters of the newborn. In addition, it is a study characterising women living in Poland; therefore, it can be used by other authors to compare women from different regions of the world. Most of the studies on insulin and IGF-1 levels are focused on women with gestational diabetes, with a primary focus on maternal health and newborn parameters. Despite the small study group, the study design can be reproduced on a larger population and extended by additional factors, and thus, the manuscript may be an inspiration for the readers.

## 2. Materials and Methods

### 2.1. Characteristics of the Study Group

Participants were 157 mother-newborn pairs of white race (Central Europa). The following inclusion criteria were applied: 18–40 years of age, trimester III of pregnancy, single pregnancy with physiological course, and delivery via planned caesarean section and good health condition confirmed by a doctor. A planned caesarean section resulted from the patient’s choice or the indications of another specialist, e.g., orthopaedic (e.g., abnormal structure of the pelvis), ophthalmologist (e.g., retinal detachment) or psychologist (fear of childbirth). Among the subjects, two age groups were distinguished: <35 and ≥35 years of age [9]. The study was conducted at the Obstetrics and Gynaecology Department of the Wroclaw Medical University, Poland. The study was conducted according to the guidelines laid down in the Declaration of Helsinki, and all procedures involving human subjects were approved by the Ethics Committee of the Faculty of Wroclaw Medical University (reference no. KB-85/2015). Written informed consent was provided by all participants.

### 2.2. Anthropometric Measurements

The respondents’ height and body weight before delivery were measured by professional personnel. The body weight before pregnancy was given by the patient. The body weight was measured using a calibrated weighing scale (accuracy ±0.1 kg), and the height was measured using a stadiometer (accuracy ±0.5 cm). The BMI (body mass index) before pregnancy was calculated based on pre-pregnancy weight (body weight reported by the patient) according to the following formula: BMI = body mass (kg)/height (m)^2^ and interpreted according to the criteria of the WHO [10]. Total gestational weight gain was estimated by subtracting the weight before pregnancy from the last measured weight before delivery and interpreted according to the guidelines of the US Institute of Medicine (IOM) [11,12]. The gestational weight gain refers to the weight that a mother gains between the time of conception and the onset of labor. Pregnant women make their first appointment at different weeks of pregnancy, and it is very difficult to determine the body weight at conception. Therefore, the pre-pregnancy body weight declared by the patient was used for the calculations. The birth weight and length of newborns were collected via clinical records. Newborn birth weight was interpreted according to the following criteria [13]: low birth weight <2500 g; normal birth weight 2500–3999 g; high birth weight ≥4000 g. Birth length was interpreted according to the following criteria [14]: short birth length <46 cm; normal birth length 46–54 cm; long birth length >54 cm. The Ponderal index (PI) was calculated according to the following formula: PI (g/cm^3^) = (birth weight (g) × 100)/birth length (cm)^3^ [15,16]. The values obtained were interpreted according to the following criteria [17]: below standard values <2.0 g/cm^3^; below standard values but acceptable 2.0–2.5 g/cm^3^; within standard values 2.5–3.0 g/cm^3^; above standard values >3.0 g/cm^3^.

### 2.3. The Food Frequency Questionnaire

The assessment of dietary preferences during pregnancy was carried out using the food frequency questionnaire (FFQ-6) validated in Poland by Niedzwiedzka et al. [18]. The FFQ-6 questionnaire is used to qualitatively assess the frequency of consumption of 62 assorted product groups divided into eight main food groups. Respondents have a choice of six food frequency options: never or almost never, once a month or less, several times a month, several times a week, every day, several times a day. The subjects were asked about the frequency of food consumption during the third trimester of pregnancy. The frequency of food consumption of each pregnant woman surveyed was converted into the daily frequency of consumption according to the criteria in Table S1.

### 2.4. Biological Material

Venous blood was collected in EDTA (edetic acid) tubes for the tests on the day the patient was admitted to the ward in the 39th week of pregnancy. Plasma was isolated from the blood and then frozen at −80 °C. In the maternal blood plasma, insulin and IGF-1 were determined using enzyme-linked immunosorbent assay (ELISA). The concentration of insulin in the maternal blood plasma was determined using the ready-made ELISA by Demeditec Diagnostic GmbH—Demeditec Insulin ELISA (catalogue no. DE2935). The concentration of IGF-1 in the maternal blood plasma was determined using the Human IGF-1 DuoSet ELISA (R&D Systems (biotechne); catalogue no. DY291-05). In order to determine the concentration of IGF-1, the plasma dilution was performed on a scale of 1:3100, following the instructions attached to the test. The readings were made on an Epoch microplate spectrophotometer by BioTek Instruments. Plasma biochemical determinations were made in the laboratory of the Department of Human Nutrition at the University of Life Sciences in Wrocław.

### 2.5. Statistics

Statistical evaluation of the results was carried out using the StatSoft STATISTICA 13.3 PL (StataCorp LP., College Station, TX, USA) computer program. The Kolmogorov–Smirnov test with the Lilliefors correction was used to check the normality of the distribution of continuous features. The following tests were used for statistical analyses: Student’s *t*-test, Tukey’s ANOVA test for different N, Mann–Whitney U test, and Kruskal–Wallis test. Multi-regression models (with logarithmic transformation where a normal distribution was not shown) were done using the weight, length, and PI of the newborn as dependent variables; insulin and IGF-1 concentration, pre-pregnancy BMI and gestational weight gain of the mother were considered as independent variables. Next, multi-regression models were done using weight, length, and PI of the newborn as dependent variables and insulin, IGF-1, and frequency of sugar and honey intake by the mother as independent variables. Statistical significance was considered at *p* < 0.05.

## 3. Results

Table 1 shows the characteristics of the studied women and newborns.

Table 2 presents the results of the frequency of consumption of selected products during the day depending on the age of the examined women. Women under 35 years of age consumed significantly more animal fats than those over 35 years of age. No statistically significant differences were found in the consumption of other products. The occurrence of statistically significant relationships between the frequency of consumption of selected groups of products and the place of residence and education of the surveyed women was also examined. Women living in rural areas ate fish, vegetables, and vegetables fats more often than women living in town (Table S2). Women with higher education consumed sweet and salty snacks significantly less often than women with primary/vocational education (Table S3). Women with secondary education consumed meat and meat products significantly more often than women with primary/vocational education (Table S3). With regard to the remaining products, no statistically significant differences were found in the consumption of product groups depending on the place of residence and education of the respondents. 

Table 3 presents the results of correlation between the frequency of consumption of selected products during the day and maternal weight gain, birth weight, and length of the newborn. The consumption of sweet or salty snacks and refined products had a significant increasing impact on weight gain during pregnancy, while consumption of whole-grain products had a decreasing impact on weight gain during pregnancy. The consumption of sweet or salty snacks, fruits, and fruit or vegetable juices during pregnancy resulted in a significant increase the birth weight of the newborn.

The concentrations of insulin and IGF-1 were determined in the blood plasma of the tested pregnant women. The median concentration of insulin in the blood plasma did not differ significantly between the two age groups (Table 4). Significant differences were found between the plasma insulin concentration in women with normal BMI (18.5–24.95 kg/m^2^) before pregnancy and those with a BMI (25.0–29.95 kg/m^2^) indicating an overweight state. No statistically significant differences were found for the remaining parameters (maternal age and weight gain and birth parameters of the newborn) in relation to insulin concentration.

**Figure 1:**
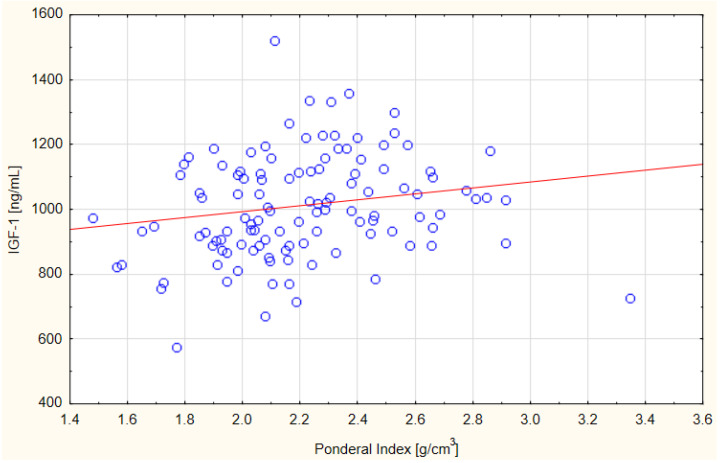
Scatter relationship between PI newborns and insulin-like growth factor 1 (IGF-1) concentration in the blood plasma of women surveyed; Spearman correlation *r* = 0.26; *p* = 0.004.

Significant differences in the concentration of IGF-1 in the blood plasma of the tested pregnant women were shown depending on the age of the women (Table 4). Women older than 35 years had significantly lower levels of plasma IGF-1 during pregnancy than women younger than 35 years. No statistically significant differences were found for the remaining parameters (maternal pre-pregnancy BMI and weight gain and birth weight and length of the newborn) in relation to IGF-1 concentration. Moreover, the increase in IGF-1 concentration was weakly positively correlated with the increase in PI in newborns (*r* = 0.26, *p* < 0.05) (Figure 1).

Over the course of the study, we tested for relationship of the frequency of consumption of selected groups of products containing carbohydrates on the concentration of insulin and IGF-1 in the blood plasma of the examined women. There were no significant associations (Table 5).

Data analysis with a multiple regression model revealed that maternal body mass index (BMI) showed a positive relationship with the newborn’s weight and Ponderal index, and the maternal IGF-1 concentration showed a positive relationship with the newborn’s Ponderal index (Table 6). Further analysis showed that the maternal frequency of honey intake only interacted with the weight of the newborn (Table 6).

## 4. Discussion

In this study, we only found that the plasma insulin concentration of the examined pregnant women differed significantly according to maternal pre-pregnancy BMI, but no association of insulin concentration and the birth parameters of newborns was found. In a study by Retnakaran et al. (2012) [19] conducted among 472 pregnant women, the relationship between the nutritional status of mothers (including anthropometric measurements, adipokines, and insulin) and the birth parameters of newborns were assessed. With regard to the insulin concentration, it was shown that the birth weight of newborns differed significantly depending on the insulin concentration in the maternal blood plasma. The highest insulin concentration (65 pmol/L) was found in women who gave birth to newborns with high birth weight, were overweight or obese before pregnancy, and experienced excessive weight gain during pregnancy. The authors also noted a significant correlation of maternal insulin and leptin levels and body fat percentage with newborn birth weight. In a pilot study by Sahasrabuddhe et al. [20] carried out among 121 pregnant women and their newborns, the impact of glucose intolerance or hyperinsulinemia in the mother during pregnancy on the concentration of insulin and glucose in the cord blood and the birth weight of children was assessed. It was shown that among mothers with pregnancies complicated by glucose intolerance or hyperinsulinemia, inhibition of intrauterine growth of the foetus was more frequent, or newborns with low birth weight were more frequent. In addition, it was shown that in the umbilical blood of the same newborns, there was a much higher concentration of insulin. The authors point to the possibility of intrauterine programming of glucose-tolerance disorders in the foetus, which may lead to the occurrence of type 2 diabetes in adulthood. This study and other research studies emphasise the importance of the nutritional status of a woman before pregnancy for the metabolism of the body during pregnancy. Increased pre-pregnancy BMI and the associated excessive growth of adipose tissue may result in increased cell resistance to this hormone during pregnancy. This condition may cause incorrectly programmed foetal carbohydrate metabolism. Insulin is responsible for the transport of nutrients to the foetus, including glucose. In pregnant women with impaired carbohydrate metabolism, there may be, for example, increased placental transport of nutrients with increased insulin resistance and, consequently, foetal macrosomia [21].

IGF-1, apart from insulin, plays an important role in the proper course of pregnancy. Its concentration increases during this period due to the growth and development of the foetus. IGF-1 is synthesized by the mother’s body and the placenta. This study showed a significant differentiation of IGF-1 concentration due to the age of the studied women and a positive weak correlation between IGF-1 and PI. In a study by Chies et al. [22] conducted among 153 pregnant women from Italy, no significant correlation was found between the concentration of IGF-1 in the mother (164 µg/L) and the birth parameters of newborns (birth weight and length and PI). In our own study, only a weak positive correlation was found between the concentration of IGF-1 in the blood plasma of the tested pregnant women and the PI of newborns. Di Biase et al. [23] assessed the relationship between IGF-1 and the prevalence of gestational diabetes. The study included 38 pregnant women with diabetes and 12 pregnant women as a control sample. IGF-1 concentration in the control group (325 ng/mL) was significantly higher than in diabetic women (279 ng/mL), while in both groups, there was no correlation with maternal anthropometric parameters. A study by Hills et al. [24] conducted among 335 pregnant women showed a significant correlation of IGF-1 concentration in mothers with their body weight before delivery, but no significant correlation with the birth weight of newborns was found. A study by Lof et al. [25] conducted among 39 Swedish pregnant women showed a significant positive correlation between the concentration of IGF-1 (398 ng/L) of mothers and their body weight before pregnancy, pregnancy weight gain, and foetal weight in the 32nd week of pregnancy. 

In a study by Verhaeghe et al. [26] conducted among 289 pregnant women, the occurrence of relationships between anthropometric measurements of the expectant mothers, the concentrations of glucose, insulin, IGF-1, Insulin Growth Factor Binding Protein-1 (IGFBP-1), and leptin in the blood and birth parameters of newborns were analysed. It was shown that the concentration of IGF-1 in the blood was determined by age, BMI, and the concentration of insulin in the blood of pregnant women. There was no significant correlation between the concentrations of insulin and IGF-1 in the mother and the birth weight of the newborn, similarly to our own research. In studies by Asvold et al. [27] conducted among 368 pregnant women, the correlation between the change in IGF-1 concentration in the blood serum of a woman in each trimester of pregnancy and the birth parameters of the newborn was assessed. The studies did not show any significant relationships between IGF-1 in the blood serum of pregnant women and birth weight and length or the Ponderal index of newborns. In the studies by Yang et al. [28] conducted among 114 pregnant women, the correlation between the concentration of IGF-1 in the blood serum of women and its anthropometric parameters was assessed. The authors showed a significant positive correlation between IGF-1 and the body weight and BMI of women. The study showed that advanced maternal age causes a decrease in growth hormone necessary for the proper growth and development of the foetus, as it directly stimulates the growth and proliferation of cells. It is worth noting that the concentration of IGF-1 peaks for women in adolescence and begins to decline after the age of 20 by about 13% for each decade. IGF-1 also has a significant effect on the birth weight of the foetus, as it is involved in the metabolism of fat and muscle tissue. Increased concentration of IGF-1 in the mother may cause excessive growth of adipose tissue in the foetus and, consequently, the occurrence of obesity in later life. The IGF-1 deficiency can cause intrauterine growth retardation [29,30].

The study showed that the women under 35 years of age consumed significantly more animal fats than those over 35 years of age. No statistically significant differences were found in the consumption of other products. The study showed that the consumption of sweet or salty snacks and grain products had a significant impact on weight gain during pregnancy, while the consumption of sweet and salty snacks, fruits, and fruit or vegetable juices determined the birth weight of the newborn. In a study by Lundqvist et al. [31] conducted among 226 pregnant women, eating habits were analysed using the FFQ questionnaire. Two age groups of women were distinguished: <35 and ≥35 years of age. It was shown that only the consumption of whole-grain products was significantly higher in older women (≥35 years of age). It was also shown that pregnant women <35 years of age consumed products that are a source of fat more often, including products rich in polyunsaturated fatty acids, and consumed products that are a source of protein less often than older women. In a study by Li et al. [32] conducted among 539 pregnant women, the influence of eating habits and physical activity on the occurrence of carbohydrate metabolism disorders was analysed, and women with diagnosed diabetes were excluded from the group. Eating habits were assessed using the FFQ questionnaire. Disorders of carbohydrate metabolism were found among 13% of women who were additionally characterized by higher body weight and greater weight gain during pregnancy and were older than women without carbohydrate metabolism disorders. Women with carbohydrate disorders were characterized by more frequent consumption of fruit with a high glycaemic index and snacks with a high energy content. A study by Zuccolotto et al. [33] conducted among 785 pregnant women assessed the impact of eating habits using the FFQ questionnaire on weight gain in pregnancy and the occurrence of carbohydrate metabolism disorders. Studies have shown that women who consumed snacks more often during pregnancy were more likely to develop overweight states, obesity, and carbohydrate disorders. A study by Coelho et al. [34] conducted among 1298 pregnant women assessed the impact of eating habits (via the FFQ questionnaire) on the birth weight of the newborn. Four eating patterns were distinguished in the study: prudent pattern (milk, yogurt, cheese, fruit and natural juice, crackers, chicken, beef, fish, and liver), traditional pattern (beans, rice, vegetables, bread, butter, margarine, and sugar), western pattern (pizza, hamburgers, deep fried pastries, potato, cassava, yams, soft drinks, flour, farofa, grits, pork, sausages, and egg), and snack pattern (sandwich cookies, salty snacks, chocolate, and chocolate drink mix). It was shown that eating patterns such as the western pattern and the snack pattern favoured the occurrence of overweight states and obesity but did not influence excessive weight gain in pregnant women. A positive correlation was also observed between the snack pattern among pregnant women and the birth weight of newborns. Similarly, in our own research, consumption of products such as sweet and salty snacks as well as fruit and fruit juices, which are a source of simple sugars, by pregnant women favoured a higher birth weight. 

This study has some limitations, including the limited sample size, which reduces the possibility of detecting other significant associations, and the subjectivity of the results. It was not possible to determine fasting glucose in all the patients, which could broaden the assessment of carbohydrate metabolism in pregnant women. Glucose concentrations were determined among a very small number of study participants; therefore, this parameter was rejected. The assessment of the frequency of consumption of products with the use of the FFQ-6 questionnaire allowed us to assess the general supply of selected groups of products in the diet from the third trimester of pregnancy. FFQ-6 is a questionnaire to assess the food consumption frequency during the year, while the authors used a questionnaire to assess the food consumption frequency from the third trimester of pregnancy. This is a certain limitation in the use of the questionnaire. This form does not show the exact supply of energy and macronutrients in the diet. It is a difficult field of research because the collection of willing pregnant women to participate in the research was difficult and associated with a large amount of diverse material. 

Despite all those potential limitations, our results shed light on the association of some maternal factors and the birth parameters of newborns. The study also shows what the hormonal balance (insulin and IGF-1) looks like in women with a physiological course of pregnancy (without gestational diabetes), while most insulin and IGF-1 studies are conducted in women with gestational diabetes.

## 5. Conclusions

In summary, the authors’ own research shows the relationship between the frequency of consumption of selected groups of products during pregnancy and weight gain in pregnancy and the birth weight of the newborn. There was no evidence of an association of the concentration of insulin and IGF-1 in the mother’s blood and the weight and birth length of the newborn; only the concentration of IGF-1 correlated with the PI of the newborn. Intensive education in healthy eating habits is essential for the proper growth and development of the foetus and the proper course of pregnancy. In the available literature, there are many reports describing the relationship of the mother’s nutrition and the health of the newborn, while there are few reports describing the relationship of the diet and the concentration of insulin and IGF-1 during pregnancy among women without gestational diabetes and the relationship of these factors and the birth parameters of the newborn. 

## Figures and Tables

**Table 1:** Basic characteristics of participants: women and newborns (*n* = 157).

Pregnant Women	
	Median (Q1; Q3)
Age (years)	31.5 (28; 34)
Body height (cm)	166.5 (159; 176)
Body weight before pregnancy (kg)	66.0 (52; 76)
Body mass index (BMI) before pregnancy (kg/m^2^)	21.7 (20.4; 25.4)
Weight gain during pregnancy (kg)	15 (10; 18)
	(%)
Place of residence	
City	73
Rural areas	27
Educational level	
Primary/vocational education	11
Secondary education	21
Higher education	68
Newborn	
	Median (Q1; Q3)
Birth length (cm)	54.0 (52; 56)
Birth weight (g)	3351 (3059; 3791)
	Average ± SD
Ponderal index (g/cm^3^)	2.2 ± 0.33
Newborn Gender	
	N
Female	65
Male	92

Q1 and Q3—first and third quartiles; N—a number of participants; SD—standard deviation.

**Table 2:** The food consumption frequency (frequency/day) of selected product groups depending on the age of surveyed pregnant women.

Product Groups	<35 Years (*n* = 137)		≥35 Years (*n* = 20)		*p*-Value
	Me	(Q1; Q3)	Me	(Q1; Q3)	
Sweet and salty snacks	2.07	(1.30; 3.28)	1.47	(0.92; 2.42)	0.076
Dairy	0.57	(0.57; 1.00)	0.57	(0.57; 1.00)	0.548
Eggs	0.57	(0.10; 0.57)	0.10	(0.10; 0.57)	0.129
Whole-grain products	1.00	(0.57; 1.00)	1.00	(0.57; 1.00)	0.803
Refined products	0.57	(0.03; 0.57)	0.10	(0.03; 1.00)	0.998
Vegetable fats	0.57	(0.2; 1.00)	0.57	(0.10; 1.00)	0.670
Animal fats	1.2	(1.00; 1.67)	1.03	(0.60; 1.20)	0.010
Fruits	1.00	(0.57; 1.00)	1.00	(1.00; 1.00)	0.770
Vegetables	1.00	(0.57; 1.00)	1.00	(0.57; 1.00)	0.232
Fruit and vegetable juices	2.67	(2.10; 3.60)	3.03	(2.10; 4.00)	0.815
Nuts and seeds	0.11	(0.02; 0.57)	0.10	(0.02; 0.57)	0.901
Meat and meat products	1.27	(0.72; 1.77)	1.33	(0.80; 1.74)	0.778
Fishes	0.13	(0.05; 0.20)	0.20	(0.10; 0.20)	0.181
Alcohol	0.00	(0.00; 0.00)	0.00	(0.00; 0.00)	0.500

Q1 and Q3—first and third quartiles.

**Table 3:** Spearman’s correlation of the food consumption frequency and maternal weight gain during pregnancy and newborn weight and length (*n* = 157).

Product Groups	Weight Gain	Birth Weight	Birth Length
Sweet and salty snacks	0.21 *	0.20 *	0.24 **
Dairy	0.05	0.08	0.03
Eggs	−0.03	0.04	−0.02
Whole-grain products	−0.21 *	0.00	−0.12
Refined products	0.20 *	0.07	0.07
Vegetable fats	0.08	0.04	0.01
Animal fats	0.10	0.03	0.02
Fruits	0.0	0.22 **	0.10
Vegetables	0.0	0.09	0.08
Fruit and vegetable juices	0.14	0.24 **	0.11
Nuts and seeds	0.07	−0.08	−0.04
Meat and meat products	0.08	0.05	−0.08
Fishes	−0.03	−0.01	−0.04
Alcohol	−0.09	0.05	0.04

* *p* < 0.05; ** *p* < 0.01.

**Table 4:** Association of plasma insulin and IGF-1 concentration in mothers with age, BMI, weight gain during pregnancy, and anthropometric measures in offspring at birth (*n* = 157).

Mothers and Neonates Parameters		Insulin (ng/mL)				IGF-1 (ng/mL)			
Mothers		*n*	Me	(Q1; Q3)	*p*-Value	*n*	Me	(Q1; Q3)	*p*-Value
Age	<35 years	137	23.5	(16.8; 32.9)	0.110	137	1098.5 ^b^	(932.1; 1118.8)	0.024
	≥35 years	20	19.6	(12.5; 28.2)		20	971.1 ^b^	(892.3; 1088.7)	
BMI	<18.5 kg/m^2^	14	20.7	(6.2; 38.0)	0.047	14	950.7	(823.0; 159.8)	0.092
	18.5–24.9 kg/m^2^	99	18.5 ^a^	(12.9; 25.2)		99	974.4	(890.0; 1118.8)	
	24.9–29.9 kg/m^2^	34	27.0 ^a^	(21.1; 36.0)		34	1048.8	(937.5; 1154.8)	
	≥30 kg/m^2^	10	23.7	(20.0; 34.6)		10	985.3	(939.1; 1101.4)	
Weight gain	Below standard	29	19.4	(11.4; 30.7)	0.212	29	1032.8	(906.1; 1176.9)	0.079
	Standard	77	20.2	(12.7; 34.0)		77	971.2	(883.1; 1115.2)	
	Above standard	51	22.4	(18.5; 27.6)		51	995.0	(926.0; 1124.9)	
Neonates									
Birth weight	<2500 g	12	22.8	(20.6; 29.7)	0.081	12	963.2	(906.1; 1162.9)	0.078
	2500–3999 g	120	20.9	(13.5; 31.1)		120	999.6	(892.7; 1118.1)	
	≥4000 g	25	22.8	(14.0; 32.0)		25	985.3	(897.3; 1114.3)	
Birth length	<46 cm	6	19.6	(10.9; 26.3)	0.053	6	816.9	(650.8; 934.6)	0.051
	46–54 cm	88	21.8	(14.3; 30.7)		88	1024.3	(908.4; 1160.1)	
	>54 cm	63	20.3	(14.4; 31.4)		63	985.9	(889.6; 1107.8)	
Ponderal index	<Standard	47	20.6	(16.3; 25.3)	0.057	47	922.8 ^c^	(832.9; 1054.7)	0.015
	<Standard, acceptable	84	22.2	(14.0; 34.6)		84	1012.8	(926.0; 1154.8)	
	Standard	26	22.0	(14.3; 27.6)		26	1034.4 ^c^	(943.0; 1118.8)	

^a^—statistically significant differences in plasma insulin concentration in mothers depending on pre-pregnancy BMI. ^b^—statistically significant differences in plasma IGF-1 concentration in mothers depending on age. ^c^—statistically significant differences in plasma IGF-1 concentration in mothers depending on ponderal index. BMI – body mass index; IGF-1—insulin-like growth factor-1; Q1 and Q3—first and third quartiles.

**Table 5:** Spearman’s correlation of the food consumption frequency and maternal insulin and IGF-1 concentrations (*n* = 157).

Food Consumption Frequency (Times Per Day)	Insulin ng/mL	IGF-1 ng/mL
Total sweet and salty snacks	−0.107	−0.187
Chocolate and chocolate products	0.024	−0.080
Salty snacks	−0.143	−0.106
Ice cream and pudding	−0.160	−0.078
Biscuits and cookies	−0.060	−0.109
Non-chocolate candies	−0.117	−0.114
Honey	−0.032	0.029
Sugar to sweeten	−0.089	−0.162
Milk and natural dairy products	0.075	0.030
Milk drinks and flavored and sweetened dairy products	0.004	0.160
Whole-meal bread	0.038	0.048
Refined bread	−0.117	−0.074
Coarse groats, brown rice, whole-meal pasta	0.066	0.065
Fine grain groats, white rice, and pasta	0.100	0.142
Breakfast products	0.079	−0.082
Fruits	0.016	0.133
Vegetables	0.179	0.090
Dry legumes	−0.004	−0.006
Potatoes	0.144	−0.095
Fruit juices	0.095	0.048
Vegetable juices	−0.011	−0.008
Sweetened carbonated drinks	−0.153	−0.124

IGF-1—insulin-like growth factor 1.

**Table 6:** Multiple regression analysis considering the weight, length, and Ponderal index of newborn (*n* = 157).

	Variable	Coefficient	*p*-Value	R^2^
Newborn weight (g)	BMI (kg/m^2^)	0.505	<0.001	0.20
	Body weight gain (kg)	0.047	0.22	
	Insulin (ng/mL)	−0.042	0.22	
	BMI (kg/m^2^)	0.451	<0.001	0.20
	Body weight gain (kg)	0.047	0.22	
	IGF-1 (ng/mL)	0.180	0.14	
	Frequency of sugar intake	−0.004	0.79	0.13
	Frequency of honey intake	0.039	0.03	
	Insulin (ng/mL)	−0.022	0.54	
	Frequency of sugar intake	−0.002	0.89	0.12
	Frequency of honey intake	0.04	0.02	
	IGF-1 (ng/mL)	0.004	0.98	
Newborn length (cm)	Height (cm)	0.071	0.07	0.04
	Body weight gain (kg)	0.009	0.47	
	Insulin (ng/mL)	−0.004	0.69	
	Height (cm)	0.067	0.08	0.03
	Body weight gain (kg)	0.009	0.48	
	IGF-1 (ng/mL)	0.001	0.97	
	Frequency of sugar intake	−0.001	0.89	0.08
	Frequency of honey intake	0.011	0.09	
	Insulin (ng/mL)	−0.009	0.54	
	Frequency of sugar intake	−0.001	0.84	0.08
	Frequency of honey intake	0.012	0.08	
	IGF-1 (ng/mL)	−0.035	0.59	
Newborn Ponderal index	BMI (kg/m^2^)	0.292	<0.001	0.13
	Body weight gain (kg)	0.018	0.47	
	Insulin (ng/mL)	−0.028	0.22	
	BMI (kg/m^2^)	0.249	0.001	0.15
	Body weight gain (kg)	0.019	0.46	
	IGF-1 (ng/mL)	0.176	0.034	
	Frequency of sugar intake	−0.001	0.92	0.002
	Frequency of honey intake	0.003	0.83	
	Insulin (ng/mL)	0.005	0.88	
	Frequency of sugar intake	0.002	0.91	0.01
	Frequency of honey intake	0.004	0.80	
	IGF-1 (ng/mL)	0.107	0.47	

BMI—body mass index; IGF-1—insulin-like growth factor 1; R^2^—coefficient of determination.

## Data Availability

The data presented in this study are available on request from the corresponding author.

## Supplementary Materials

The following are available online at https://www.mdpi.com/article/10.3390/nu13072344/s1, Table S1: Food consumption frequency categories and daily consumption frequency used in the questionnaire, Table S2: The food consumption frequency (frequency/day) of selected product groups depending on the place of residence of pregnant women, Table S3: The food consumption frequency (frequency/day) of selected product groups depending on the education of pregnant women.
