# Supplementary material for: Circulating Insulin and IGF-1 and Frequency of Food Consumption during Pregnancy as Predictors of Birth Weight and Length
Source: Nutrients. 2021 Jul 9;13(7):2344. doi: 10.3390/nu13072344 (PMC8308892; doi:10.3390/nu13072344)
Supplement: Supplementary file 1 [file nutrients-13-02344-s001.zip › nutrients-1262308-supplementary.pdf]

Supplementary tables

Table S1. Food consumption frequency categories and daily consumption frequency used in the questionnaire**.**

The frequency of consumption	Daily consumption frequency rate
Never/almost never	0
Once a month or less	0.025
Several times a month	0.1
A few times a week	0.571
Every day	1
Several times a day	2

Table S2. The food consumption frequency (frequency/day) of selected product groups depending on the place of residence of pregnant women.

Product groups	Town (n = 115)		Rural areas (n = 42)		p-value
	Me	(Q1;Q3)	Me	(Q1;Q3)	
Sweet and salty snacks	2.05	1.23;2.97	2,26	1.38;4.48	0.20
Dairy	0.57	0.57.1.00	0,79	0.57;1.00	0.89
Eggs	0.57	0.10;0.57	0,57	0.10;0.57	0.94
Whole grain products	1.00	0.57;1.00	0,34	0.00;1.00	0.14
Refined products	0.10	0.10;0.57	0,57	0.00;1.00	0.10
Vegetable fats	0.57	0.13;0.67	0,63	0.57;1.14	0.038
Animal fats	1.13	1.00;1.67	1,18	0.62;1.67	0.77
Fruits	1.00	0.57;1.00	1,00	0.57;1.00	0.25
Vegetables	0.57	0.57;1.00	1,00	0.57;2.00	0.039
Fruit and vegetable juices	2.67	2.10;3.57	2,62	1.81;3.57	0.25
Nuts and seeds	0.03	0.20;0.57	0.02	0.00;0.57	0.54
Meat and meat products	1.25	2.10;3.57	1,40	0.82;1.81	0.56
Fishes	0.13	0.05;0.20	0,20	0.10;0.20	0.03
Alcohol	0.00	0.00;0.00	0,00	0.00;0.03	0.63

Table S3. The food consumption frequency (frequency/day) of selected product groups depending on the education of pregnant women.

Product groups	Higher education (n = 107)		Secondary education (n = 33)		Primary/ vocational education (n = 17)		p-value
	Me	(Q1;Q3)	Me	(Q1;Q3)	Me	(Q1;Q3)	
Sweet and salty snacks	1.82	0.92; 2,81	2.42	1.37;3.89	3.42	0.99;3.34	0.02
Dairy	0.57	0.57; 1,00	0.57	0.57;1.00	0.57	0.10;0.57	0.41
Eggs	0.57	0.10; 0,57	0.10	0.10;0.57	0.57	0.100.57	0.64
Whole grain products	1.00	0.57;1.00	0.57	0.10;1.00	1.00	0.57;2.00	0.20
Refined products	0.10	0.03;0.57	0.57	0.03;1.00	0.03	0.00;0.57	0.31
Vegetable fats	0.57	0.13;1.00	0.67	0.57;1.00	1.00	0.03;1.00	0.24
Animal fats	1.13	0.69;1.59	1.17	0.77;1.67	1.03	0.13;2.10	0.54
Fruits	1.00	0.57;1.00	1.00	0.57;1.00	1.00	0.10;2.00	0.81
Vegetables	1.00	0.57;1.00	1.00	0.57;1.00	1.00	0.57;2.00	0.99
Fruit and vegetable juices	2.59	2.10;3.57	2.71	2.20;3.57	2.00	0.87;4.2	0.31
Nuts and seeds	0.20	0.10;0.57	0.10	0.00;0.20	0.00	0.00;0.03	0.89
Meat and meat products	1.24	0.75;1.76	1.26	0.72;1.7	0.57	0.20;0.69	0.02
Fishes	0.20	0.050.20	0.20	0.10;0.20	0.05	0.05;0.13	0.44
Alcohol	0.00	0.00;0.00	0.00	0.00;0.00	0.00	0.00;0.03	0.67
